# Even and Odd Cat States of Two and Three Qubits in the Probability Representation of Quantum Mechanics

**DOI:** 10.3390/e26060485

**Published:** 2024-05-31

**Authors:** Matyas Mechler, Margarita A. Man’ko, Vladimir I. Man’ko, Peter Adam

**Affiliations:** 1 Institute for Solid State Physics and Optics, HUN-REN Wigner Research Centre for Physics, P.O. Box 49, H-1525 Budapest, Hungary; mechler.matyas@wigner.hun-ren.hu; 2 Institute of Physics, University of Pécs, Ifjúság Útja 6, H-7624 Pécs, Hungary; 3 Lebedev Physical Institute, Leninskii Prospect 53, Moscow 119991, Russia; mankoma@lebedev.ru (M.A.M.); mankovi@lebedev.ru (V.I.M.)

**Keywords:** probability distribution, dequantizer and quantizer operators, symplectic tomography, even and odd cat states, entanglement

## Abstract

We derive the probability representation of even and odd cat states of two and three qubits. These states are even and odd superpositions of spin-1/2 eigenstates corresponding to two opposite directions along the *z* axis. The probability representation of even and odd cat states of an oscillating spin-1/2 particle is also discussed. The exact formulas for entangled probability distributions describing density matrices of all these states are obtained.

## 1. Introduction

Recently, the probability representation of quantum states was suggested for systems with both continuous and discrete variables [1,2,3,4,5,6,7,8]. In this representation, the system states were expressed through regular nonnegative probability distributions defined in the phase space. These probability distributions can be derived from the density operator, and they contain all information on the quantum system. The probability representation can be used to explain all quantum effects effectively by using the standard properties of the conventional probability theory. The probability representation is related to other quasiprobability representations of quantum states, such as the Glauber–Sudarshan *P*-function [9,10], the Husimi *Q*-function [11,12], and the Wigner function [13] by integral transforms [14]. For deriving probability representations, a general formalism based on dequantizer and quantizer operators that describes all invertible maps associating operators acting on a Hilbert space and functions of certain variables has also been developed [15].

A widely studied type of probability representation of continuous quantum systems is the symplectic tomogram. For special sets of parameters, this tomogram coincides with the optical homodyne tomogram [6] that can be measured in quantum optical experiments [16]. Optical tomograms can be applied to reconstruct the density matrix and the Wigner function [17,18,19,20,21]. Recently, probability representations called symplectic tomograms have been determined for several important states of the harmonic oscillator, including thermal states [22], coherent states, Fock states [8], and Schrödinger cat states [23], which were originally introduced under the name even and odd coherent states. The time evolution of these tomograms, initially prepared in the potential of the usual harmonic oscillator, has also been derived for free particle motion [6,8,22,23] or for inverted oscillators [24,25,26].

The probability representations of quantum systems with discrete variables have also been developed and applied for qubit and qudit systems [2,3,27,28,29,30]. In Refs. [31,32], probability distributions for one- and two-qubit states have been introduced where the components of the distributions correspond to the probabilities of various spin projections onto the opposite directions of the perpendicular *x*, *y*, and *z* axes for the particular qubits. For two-qubit entangled states, such probability distributions are entangled ones that can be distinguished from distributions of separable states [31,32]. The physical relevance of these types of discrete probability representations is that the components of the probability distributions can be easily measured experimentally by repeated spin projection measurements using a sufficiently large set of identically prepared states. Then, the elements of the density matrix can be expressed by the components of these representations [31]. Consequently, the time evolution and any quantum effect can be treated by using these measurable probabilities.

In this paper, we derive the previously discussed probability representation for even and odd cat states of two and three qubits, that is, even and odd superpositions of spin-1/2 eigenstates corresponding to opposite directions along the *z*-axis for the particular qubits. In the case of two qubits, they correspond to two of the Bell states [33,34,35], while for three qubits, the even cat state is equal to the GHZ state [36,37]. These entangled states have several applications in quantum information technology [38,39,40,41,42]. One of our aims is to determine the components of the entangled conditional probability distributions of two-qubit even and odd cat states and that of three-qubit even cat states and also the components of the marginal conditional probability distributions. We also discuss the probability representation of even and odd cat states of an oscillating spin-1/2 particle. Our motivation to discuss these states is that the even and odd cat states of different oscillator systems, known as Schrödinger cat states [43,44,45], can be produced in various experiments [46,47,48,49,50,51,52,53,54], and they can have several applications in quantum information processing schemes e.g., in optical quantum computation [55,56,57,58] and quantum communication [59,60]. The development of schemes for the generation and application of the considered cat states of oscillating spin-1/2 particles can be anticipated. We introduce an entangled conditional probability distribution with both a continuous and a discrete variable concerning the oscillator and the spin-1/2 states, respectively, and we derive all the factors required to determine the entangled conditional probability distributions corresponding to the density matrices of these states. We also define marginal conditional probability distributions characterizing the state of either the spin or the oscillator, and we determine these distributions. We evaluate the characteristics of all the derived probability distributions and clarify the way the entanglement appears in their measurable components.

This paper is organized as follows.

In Section 2, we review the construction of probability representation of quantum states both for continuous and discrete variables. In Section 3, we present our results on the probability representation of even and odd cat states of two and three qubits and also on that of even and odd cat states of an oscillating spin-1/2 particle. Finally, we summarize our findings and draw conclusions in Section 4.

## 2. Probability Representations and the Formalism of Dequantizers and Quantizers

### 2.1. Continuous Dimensional Quantum Systems

The probability representation of quantum states can be derived by using the formalism of dequantizer operators $\hat{U}\left(\bar{x}\right)$ and quantizer operators $\hat{D}\left(\bar{x}\right)$ [15]. The parameter $\bar{x}$ labels the particular operators, and it can have either discrete or continuous components $x_{1},x_{2},\cdots ,x_{n}$. The operators $\hat{U}\left(\bar{x}\right)$ and $\hat{D}\left(\bar{x}\right)$ can be used to construct an invertible map of operators $\hat{A}$ acting on the Hilbert space H onto functions $f_{A}\left(\bar{x}\right)$ via the formulas [15]

(1) $$\begin{array}{ccc} f_{A}\left(\bar{x}\right) & = & \mathrm{Tr}(\hat{A}\hat{U}\left(\bar{x}\right)), \end{array}$$

(2) $$\begin{array}{ccc} \hat{A} & = & \int f_{A}\left(\bar{x}\right)\hat{D}\left(\bar{x}\right)\;d\bar{x}. \end{array}$$

The function $f_{A}\left(\bar{x}\right)$ is the symbol of the operator $\hat{A}$. In the case of discrete variables $x_{i}$, the integral in this equation should be replaced by a corresponding sum:

(3) $$\begin{array}{ccc} f_{A}^{\left(i\right)} & = & \mathrm{Tr}\left(\hat{A}{\hat{U}}^{\left(i\right)}\right),\;i=1,\ldots ,l, \end{array}$$

(4) $$\begin{array}{ccc} \hat{A} & = & \sum_{i=1}^{n}f_{A}^{\left(i\right)}{\hat{D}}_{A}^{\left(i\right)}. \end{array}$$

Expressions (1) to (4) are valid whenever $\hat{A}$ is a density operator. Using the dequantizer operator $\hat{U}\left(X,\mu ,\nu \right)=\delta \left(X\hat{1}-\mu \hat{q}-\nu \hat{p}\right)$, the density operator $\hat{\rho }$ of a continuous-variable quantum system can be mapped onto the function w(X∣μ,ν) known as symplectic tomogram by the expression [1]

(5) $$w\left(X|\mu ,\nu \right)=\mathrm{Tr}\left[\hat{\rho }\;\delta \left(X\hat{1}-\mu \hat{q}-\nu \hat{p}\right)\right],$$

where $\hat{q}$ and $\hat{p}$ are the position and momentum operators, respectively. The function w(X∣μ,ν) is a nonnegative conditional probability distribution function of the random position *X*. This distribution satisfies the normalization condition

(6) ∫w(X∣μ,ν) dX=1.

The conditions are labeled by parameters μ and ν in the reference frame where the position *X* is measured; that is, the position *X* is determined as X=μq+νp in the phase space. By applying the quantizer operator $\hat{D}\left(X,\mu ,\nu \right)=\exp\left[i\left(X\hat{1}-\mu \hat{q}-\nu \hat{p}\right)\right]$, the inverse transformation can be obtained as

(7) $$\hat{\rho }=\frac{1}{2\pi }\int w\left(X|\mu ,\nu \right)\exp\left[i\left(X\hat{1}-\mu \hat{q}-\nu \hat{p}\right)\right]\;dX\;d\mu \;d\nu .$$

For pure states $\hat{\rho }=\left|\psi \rangle \langle \psi \right|$, expression (5) can be converted into the formula [8]

(8) $$w\left(X|\mu ,\nu \right)=\frac{1}{2\pi |\nu |}{\left|\int \psi \left(y\right)\exp\left(\frac{i\mu }{2\nu }y^{2}-\frac{iX}{\nu }y\right)\;dy\right|}^{2},$$

where ψ(y)=〈y||ψ〉 is the wave function of the state.

The symplectic tomogram can also be derived from the Wigner function W(q,p) of the state by using the expression

(9) $$w\left(X|\mu ,\nu \right)=\frac{1}{2\pi }\int W\left(q,p\right)\delta \left(X-\mu q-\nu p\right)\;dq\;dp.$$

The Wigner function can be derived from the density operator as [8]

(10) $$W\left(q,p\right)=\frac{1}{2\pi }\int_{-\infty }^{\infty }\langle q-u/2|\hat{\rho }|q+u/2\rangle e^{ipu}\;du,$$

and it can be obtained from the symplectic tomogram as [8]

(11) $$W\left(q,p\right)=\frac{1}{2\pi }\int w\left(X|\mu ,\nu \right)e^{i(X-\mu q-\nu p)}\;dX\;d\mu \;d\nu .$$

The symplectic tomogram w(X∣μ,ν) and the Wigner function W(q,p) contain all information on the density operator; that is, they can be used to completely characterize the quantum state. As was stated before, symplectic tomograms are always nonnegative functions; consequently, they are regular probability distributions. In contrast, in the case of Wigner functions, the occurrence of negative values is quite common and indicates the presence of quantum interference.

### 2.2. Finite Dimensional Quantum Systems

For a finite *d*-dimensional quantum system, the minimal set of dequantizer operators comprises $d^{2}$ elements. For example, in the case of qubits, that is, for d=2, a possible minimal set of dequantizer operators is

(12) $$U^{\left(1\right)}=\frac{1}{2}\left(\begin{array}{cc} 1 & 1\\ 1 & 1 \end{array}\right),\;U^{\left(2\right)}=\frac{1}{2}\left(\begin{array}{cc} 1 & -i\\ i & 1 \end{array}\right),\;U^{\left(3\right)}=\left(\begin{array}{cc} 1 & 0\\ 0 & 0 \end{array}\right),\;U^{\left(4\right)}=\left(\begin{array}{cc} 0 & 0\\ 0 & 1 \end{array}\right),$$

and the corresponding quantizers are

(13) $$D^{\left(1\right)}=\left(\begin{array}{cc} 0 & 1\\ 1 & 0 \end{array}\right),\;D^{\left(2\right)}=\left(\begin{array}{cc} 0 & -i\\ i & 0 \end{array}\right),\;D^{\left(3\right)}=\left(\begin{array}{cc} 1 & \frac{-1+i}{2}\\ \frac{-1-i}{2} & 0 \end{array}\right),\;D^{\left(4\right)}=\left(\begin{array}{cc} 0 & \frac{-1+i}{2}\\ \frac{-1-i}{2} & 1 \end{array}\right).$$

These dequantizer and quantizer operators satisfy the orthogonality condition

(14) $$\mathrm{Tr}\left({\hat{D}}^{\left(i\right)}{\hat{U}}^{\left(j\right)}\right)=\delta_{ij},\;i,j=1,\ldots ,4.$$

Applying Equation (3), one can obtain the probability representation of qubit states as

(15) $$\mathrm{Tr}\left(\hat{\rho }U^{\left(i\right)}\right)=p_{i},\;i=1,2,3;\;\mathrm{Tr}\left(\hat{\rho }U^{\left(4\right)}\right)=p_{4}=1-p_{3}.$$

Then, the density operator of the qubit state can be obtained by applying Equation (4),

(16) $$\hat{\rho }=\sum_{i=1}^{4}p_{i}{\hat{D}}^{\left(i\right)}$$

which yields

(17) $$\hat{\rho }=\left(\begin{array}{cc} p_{3} & \left(p_{1}-1/2\right)-i\left(p_{2}-1/2\right)\\ \left(p_{1}-1/2\right)+i\left(p_{2}-1/2\right) & 1-p_{3} \end{array}\right).$$

Accordingly, the elements of the density operator are determined by measurable probabilities. Taking into account the nonnegativity of the density operator $\hat{\rho }$, the following constraints can be found for the probabilities $p_{1}$, $p_{2}$, $p_{3}$:

(18) $${\left(p_{1}-\frac{1}{2}\right)}^{2}+{\left(p_{2}-\frac{1}{2}\right)}^{2}+{\left(p_{3}-\frac{1}{2}\right)}^{2}\leq \frac{1}{4}.$$

The physical meaning of this probability representation can be revealed by introducing the conditional probability distribution w(X∣j), where the parameter *X* can take values only from the set {+1/2,−1/2} while parameter *j* can take values from the set {1,2,3}. The particular components of this distribution correspond to the probabilities of the spin projection X=±1/2 onto the perpendicular *x* (j=1), *y* (j=2), and *z* (j=3) directions as follows

(19) $$\begin{array}{ccc} w(+1/2|1)=p_{1}, & \;w(+1/2|2)=p_{2}, & \;w(+1/2|3)=p_{3},\\ w(-1/2|1)=1-p_{1}, & \;w(-1/2|2)=1-p_{2}, & \;w(-1/2|3)=1-p_{3}. \end{array}$$

This conditional probability distribution obviously satisfies the condition

(20) $$\sum_{X}w\left(X|j\right)=1,\;j=1,2,3.$$

The dequantizer operators determining the distribution w(X∣j) are the density operators corresponding to the six normalized eigenvectors of the Pauli matrices ${\hat{\sigma }}_{x}$, ${\hat{\sigma }}_{y}$, ${\hat{\sigma }}_{z}$, which describe the spin projections X=±1/2 on the three axes [32]:

(21) $$\begin{array}{cc} \hat{U}\left(+1/2|1\right)=\frac{1}{2}\left(\begin{array}{cc} 1 & 1\\ 1 & 1 \end{array}\right), & \;\hat{U}\left(-1/2|1\right)=\frac{1}{2}\left(\begin{array}{cc} 1 & -1\\ -1 & 1 \end{array}\right),\\ \hat{U}\left(+1/2|2\right)=\frac{1}{2}\left(\begin{array}{cc} 1 & -i\\ i & 1 \end{array}\right), & \;\hat{U}\left(-1/2|2\right)=\frac{1}{2}\left(\begin{array}{cc} 1 & i\\ -i & 1 \end{array}\right),\\ \hat{U}\left(+1/2|3\right)=\left(\begin{array}{cc} 1 & 0\\ 0 & 0 \end{array}\right), & \;\hat{U}\left(-1/2|3\right)=\left(\begin{array}{cc} 0 & 0\\ 0 & 1 \end{array}\right). \end{array}$$

Note that four of these dequantizers coincide with the dequantizers of the minimal set given in Equation (12), namely, $\hat{U}\left(+1/2|1\right)={\hat{U}}^{\left(1\right)}$, $\hat{U}\left(+1/2|2\right)={\hat{U}}^{\left(2\right)}$, $\hat{U}\left(+1/2|3\right)={\hat{U}}^{\left(3\right)}$, and $\hat{U}\left(-1/2|3\right)={\hat{U}}^{\left(4\right)}$.

Applying the dequantizers in Equation (21), the conditional probabilities w(X∣j) in Equation (19) can be obtained as

(22) $$\begin{array}{c} w\left(X|j\right)=\mathrm{Tr}\left[\hat{\rho }\hat{U}\left(X|j\right)\right],\;X=\pm \frac{1}{2},\;j=1,2,3. \end{array}$$

These probabilities contain all information about the density operator of the qubit state. For example, the matrix elements of the density matrix can be expressed by applying Equations (17) and (19):

(23) $$\begin{array}{c} \rho_{1,1}=w(+1/2|3),\\ \rho_{2,1}=(w(+1/2|1)-1/2)+i(w(+1/2|2)-1/2),\\ \rho_{1,2}=(w(+1/2|1)-1/2)-i(w(+1/2|2)-1/2),\\ \rho_{2,2}=w(-1/2|3). \end{array}$$

The unitary transform of the spin state density operators, including the unitary time evolution of the system, can be also treated in this probability representation [32].

## 3. Results

In this section we present our results on the probability representations for two- and three-qubit cat states and also for cat states of an oscillating spin-1/2 particle.

### 3.1. Qubit Cat States

First, let us derive the probability representation of qubit even and odd cat states

(24) $$|\psi_{\mathrm{cat}}^{\pm }\rangle =\frac{|0\rangle \pm |1\rangle }{\sqrt{2}}$$

in order to show how the method works. The corresponding density operator is

(25) $${\hat{\rho }}_{\mathrm{cat},1}^{\pm }=|\psi_{\mathrm{cat}}^{\pm }\rangle \langle \psi_{\mathrm{cat}}^{\pm }|=\frac{1}{2}\left(|0\rangle \langle 0|\pm |0\rangle \langle 1|\pm |1\rangle \langle 0|+|1\rangle \langle 1|\right)$$

which can be expressed in the computational basis $|0\rangle =\left(\begin{array}{c} 1\\ 0 \end{array}\right)$, $|1\rangle =\left(\begin{array}{c} 0\\ 1 \end{array}\right)$ as

(26) $$\rho_{\mathrm{cat},1}^{\pm }=\frac{1}{2}\left(\begin{array}{cc} 1 & \pm 1\\ \pm 1 & 1 \end{array}\right).$$

The conditional probabilities $w_{\mathrm{cat},1}^{\pm }\left(X|j\right)$ can be derived using Equations (21) and (22), which leads to the values presented in Table 1.

The probabilities in Table 1 contain all information about qubit even and odd cat states.

### 3.2. Two-Qubit Cat States

The even and odd cat states for two qubits can be defined as

(27) $$|\Phi_{\mathrm{cat},2}^{\pm }\rangle =\frac{|00\rangle \pm |11\rangle }{\sqrt{2}}.$$

Note that these two states correspond to two of the Bell states. The case of even cat states for two qubits was also discussed in Ref. [32]. Note that these states are entangled states that cannot be written in the form of the product of the states of two qubits. The density operator corresponding to the two-qubit cat states can be written as

(28) $${\hat{\rho }}_{\mathrm{cat},2}^{\pm }=|\Phi_{\mathrm{cat},2}^{\pm }\rangle \langle \Phi_{\mathrm{cat},2}^{\pm }|=\frac{1}{2}\left(|00\rangle \langle 00|\pm |00\rangle \langle 11|\pm |11\rangle \langle 00|+|11\rangle \langle 11|\right).$$

Expressing the density matrix in the computational basis leads to

(29) $$\rho_{\mathrm{cat},2}^{\pm }=\frac{1}{2}\left(\begin{array}{cccc} 1 & 0 & 0 & \pm 1\\ 0 & 0 & 0 & 0\\ 0 & 0 & 0 & 0\\ \pm 1 & 0 & 0 & 1 \end{array}\right).$$

Two-qubit states can be characterized by the conditional probability distribution w(X,Y∣j,k) with X,Y=±1/2 and j,k=1,2,3 satisfying the normalization condition

(30) $$\sum_{X}\sum_{Y}w\left(X,Y|j,k\right)=1.$$

To obtain the components of this distribution, the dequantizer operators can be defined as

(31) $$\hat{U}\left(X,Y|j,k\right)=\hat{U}\left(X|j\right)⊗\hat{U}\left(Y|k\right),$$

where X=+1/2,−1/2, Y=+1/2,−1/2, j=1,2,3, k=1,2,3. By applying these dequantizers, the components of the conditional probability distribution w(X,Y∣j,k) can be obtained as

(32) $$\begin{array}{c} w\left(X,Y|j,k\right)=\mathrm{Tr}\left[\hat{\rho }\hat{U}\left(X,Y|j,k\right)\right]. \end{array}$$

Applying Equations (31) and (32) to the density operators ${\hat{\rho }}_{\mathrm{cat},2}^{\pm }$ of the even and odd cat states for two qubits, one can obtain the conditional probability distributions $w_{\mathrm{cat},2}^{+}\left(X,Y|j,k\right)$ and $w_{\mathrm{cat},2}^{-}\left(X,Y|j,k\right)$ presented in Table A1 and Table A2, respectively. It can be easily verified that the components of the probability distributions in these tables satisfy the normalization condition in Equation (30). The general formulae for obtaining the density matrix of two-qubit states from the considered probability representation can be found in Ref. [31]. We have also checked that the density operator in Equation (29) can be reconstructed by using these formulae and the components of the derived probability distribution displayed in Table A1 and Table A2.

Recall that the particular components of these distributions correspond to the probabilities of the spin projection X=±1/2 for the first qubit and Y=±1/2 for the second qubit onto the perpendicular *x*, *y*, and *z* directions. Hence, the zero conditional probabilities in Table A1 and Table A2 correspond to the spin-measurement outputs that cannot occur. In particular, measuring the spin in the *z* direction for both qubits the output cannot be opposite; that is, the probabilities $w_{\mathrm{cat},2}^{\pm }\left(+1/2,-1/2|3,3\right)$ and $w_{\mathrm{cat},2}^{\pm }\left(-1/2,+1/2|3,3\right)$ are equal to zero. These results can be easily understood as the two-qubit even and odd cat states are defined in the computational bases of the two qubits determined by the eigenstates of the spin measurements in the *z* direction, and both terms in these entangled states that are |00〉 and |11〉 correspond to two-qubit states with identical spin. Measuring both spins in the *x* direction (j=k=1), the measurement results cannot be opposite (identical) for the two-qubit even (odd) cat states, respectively. In contrast, measuring both spins in the *y* direction (j=k=2), the measurement results cannot be identical (opposite) for the two-qubit even (odd) cat states, respectively. These latter findings cannot be easily deduced; they represent the physical properties of the entanglement in the two-qubit even and odd cat state. To elucidate this in more detail, we will compare the entangled probability distribution of the pure even cat state with the probability distribution of the corresponding mixed states at the end of the next section.

In the case of two qubits, the marginal conditional probability distributions $\tilde{w}\left(X|j\right)$ and $\tilde{w}\left(Y|k\right)$ concerning the states of the particular qubits can be calculated from the two-qubit probability distribution as

(33) $$\begin{array}{ccc} \tilde{w}\left(X|j\right) & = & \sum_{Y}w\left(X,Y|j,k\right), \end{array}$$

(34) $$\begin{array}{ccc} \tilde{w}\left(Y|k\right) & = & \sum_{X}w\left(X,Y|j,k\right). \end{array}$$

These expressions are valid for any values of *j* and *k*. The components of the marginal conditional probability distributions ${\tilde{w}}_{\mathrm{cat},2}^{\pm }\left(X|j\right)$ and ${\tilde{w}}_{\mathrm{cat},2}^{\pm }\left(Y|k\right)$ of two-qubit even and odd cat states are displayed in Table 2.

As shown in the table, the components of the marginal conditional probability distribution ${\tilde{w}}_{\mathrm{cat},2}^{\pm }\left(X|j\right)$ are the same as ${\tilde{w}}_{\mathrm{cat},2}^{\pm }\left(Y|k\right)$. Also, the components of the conditional probability distributions are the same for even and odd cat states; that is, ${\tilde{w}}_{\mathrm{cat},2}^{+}={\tilde{w}}_{\mathrm{cat},2}^{-}$ for both particular qubits. The physical reason is that both probability distributions correspond to the mixed state that can be obtained by taking the partial trace of the state (28) for any of the qubits, that is,

(35) $${\hat{\rho }}^{\pm }={\mathrm{Tr}}_{1}\left[{\hat{\rho }}_{\mathrm{cat},2}^{\pm }\right]={\mathrm{Tr}}_{2}\left[{\hat{\rho }}_{\mathrm{cat},2}^{\pm }\right]=\frac{\left|0\rangle \langle 0|+|1\rangle \langle 1\right|}{2}.$$

By comparing the results presented in Table 1 and Table 2, one can deduce the difference between the probability representation of the superposition state defined in Equation (24) and that of the mixed state presented in Equation (35). Measuring the spin in any direction for the mixed state, the probabilities of obtaining +1/2 or −1/2 are the same, that is, 0.5. In contrast, in Table 1, the probability of obtaining +1/2 is 1 for the qubit even cat state, and, accordingly, it is 0 for the odd cat state, while the probabilities of obtaining −1/2 are 0 and 1 for the qubit even and odd cat states, respectively. This is due to the fact that the even cat state is the eigenstate of the Pauli matrix $\sigma_{x}$ corresponding to the spin projection onto the *x* direction with the eigenvalue +1/2, while the odd cat state is the other eigenstate of $\sigma_{x}$ with the eigenvalue −1/2.

### 3.3. Three-Qubit Cat States

The concepts and procedure presented for one- and two-qubit states can be extended to corresponding states of more than two qubits. For example, even and odd cat states for three qubits can be defined as

(36) $$|\Psi_{\mathrm{cat},3}^{\pm }\rangle \;=\frac{|000\rangle \pm |111\rangle }{\sqrt{2}}.$$

Note that the state $|\Psi_{\mathrm{cat},3}^{+}\rangle$ corresponds to the three-qubit Greenberger–Horne–Zeilinger state (GHZ state). Also, the states of Equation (36) are entangled states that cannot be written in the form of the product of the states of three qubits. The density operator corresponding to these states are

(37) $${\hat{\rho }}_{\mathrm{cat},3}^{\pm }=\;|\Psi_{\mathrm{cat},3}^{\pm }\rangle \langle \Psi_{\mathrm{cat},3}^{\pm }|=\frac{1}{2}\left(|000\rangle \langle 000|\pm |000\rangle \langle 111|\pm |111\rangle \langle 000|+|111\rangle \langle 111|\right)$$

and the corresponding density matrix in the computational basis can be written as

(38) $$\begin{array}{ccc} \rho_{\mathrm{cat},3}^{\pm } & = & \frac{1}{2}\left(\begin{array}{cccccccc} 1 & 0 & 0 & 0 & 0 & 0 & 0 & \pm 1\\ 0 & 0 & 0 & 0 & 0 & 0 & 0 & 0\\ 0 & 0 & 0 & 0 & 0 & 0 & 0 & 0\\ 0 & 0 & 0 & 0 & 0 & 0 & 0 & 0\\ 0 & 0 & 0 & 0 & 0 & 0 & 0 & 0\\ 0 & 0 & 0 & 0 & 0 & 0 & 0 & 0\\ 0 & 0 & 0 & 0 & 0 & 0 & 0 & 0\\ \pm 1 & 0 & 0 & 0 & 0 & 0 & 0 & 1 \end{array}\right). \end{array}$$

Similarly to one- and two-qubit states, three-qubit states can be characterized by the conditional probability distribution w(X,Y,Z∣j,k,l) with X,Y,Z=±1/2 and j,k,l=1,2,3 satisfying the normalization condition

(39) $$\sum_{X}\sum_{Y}\sum_{Z}w\left(X,Y,Z|j,k,l\right)=1.$$

To derive the components of this distribution, the following dequantizer operators can be applied:

(40) $$\hat{U}\left(X,Y,Z|j,k,l\right)=\hat{U}\left(X|j\right)⊗\hat{U}\left(Y|k\right)⊗\hat{U}\left(Z|l\right),$$

where X=+1/2,−1/2, Y=+1/2,−1/2, Z=+1/2,−1/2, j=1,2,3, k=1,2,3, l=1,2,3. Applying these dequantizers, the components of the conditional probability distribution w(X,Y,Z∣j,k,l) can be defined as

(41) $$\begin{array}{c} w\left(X,Y,Z|j,k,l\right)=\mathrm{Tr}\left[\hat{\rho }\hat{U}\left(X,Y,Z|j,k,l\right)\right]. \end{array}$$

As an example, we calculate the conditional probability distribution $w_{\mathrm{cat},3}^{+}\left(X,Y,Z|j,k,l\right)$ for the even cat state $|\Psi_{\mathrm{cat},3}^{+}\rangle$ using Equations (40) and (41). Note that this involves 216 probabilities; therefore, we present the results in six tables (Table A3, Table A4, Table A5, Table A6, Table A7 and Table A8) corresponding to different pairs of the values of the parameters *X* and *j*. One can check that the components of the probability distribution $w_{\mathrm{cat},3}^{+}\left(X,Y,Z|j,k,l\right)$ in these tables satisfy the normalization condition in Equation (39).

In accordance with the two-qubit case, the zero conditional probabilities correspond to the spin-measurement outputs that cannot occur. Similarly to the findings for the two-qubit even and odd cat states, when measuring the spin in the *z* direction for all three qubits (j=k=l=3) or for any pairs of qubits (j=k=3≠l, j=l=3≠k, or k=l=3≠j), the measurement results should be identical; that is, only 100 conditional probabilities corresponding to this criterion differ from zero, while 30 probabilities are zero in Table A3, Table A4, Table A5, Table A6, Table A7 and Table A8. The physical reason for this observation is similar to the one in the two-qubit case: both terms in the three-qubit entangled states, i.e., |000〉 and |111〉, correspond to three-qubit states in the computational bases with identical spins in the *z*-direction. The result of the measurement in this direction can be either +1/2 or −1/2 for any of the spins; however, assuming one of the outputs the measurement of the other spins can only lead to the same result according to the rules of quantum mechanics.

The other 16 zeros in the tables correspond to the following impossible measurement outputs. First, when performing measurements in the *x* direction for all three qubits, the measurement outputs cannot all be −1/2. Having a single −1/2 measurement result for one of the qubits is also excluded. Second, performing measurements in the *x* direction for one of the qubits and in the *y* direction for the other two qubits can yield two different results depending on the outcome of the measurement in the *x* direction. If the outcome of the measurement in the *x* direction is +1/2, then the outcomes of the other two measurements can be either +1/2 or −1/2, but these outcomes are identical. If the outcome of the *x*-measurement is −1/2, then the outcomes of the other two measurements are opposite. These observations cannot be explained straightforwardly; they are special consequences of the entanglement characterizing the three-qubit even cat state.

In the case of three qubits, the marginal conditional probability distributions $\tilde{w}\left(X|j\right)$, $\tilde{w}\left(Y|k\right)$, and $\tilde{w}\left(Z|l\right)$ concerning the particular qubits can be calculated from the three-qubit probability distribution as follows:

(42) $$\begin{array}{ccc} \tilde{w}\left(X|j\right) & = & \sum_{Y,Z}w\left(X,Y,Z|j,k,l\right), \end{array}$$

(43) $$\begin{array}{ccc} \tilde{w}\left(Y|k\right) & = & \sum_{X,Z}w\left(X,Y,Z|j,k,l\right), \end{array}$$

(44) $$\begin{array}{ccc} \tilde{w}\left(Z|l\right) & = & \sum_{X,Y}w\left(X,Y,Z|j,k,l\right). \end{array}$$

These expressions are valid for any values of *j*, *k*, and *l*. One can easily check that in the case of even and odd cat states, the marginal conditional probability distributions ${\tilde{w}}_{\mathrm{cat},3}^{\pm }\left(X|j\right)$, ${\tilde{w}}_{\mathrm{cat},3}^{\pm }\left(Y|k\right)$, and ${\tilde{w}}_{\mathrm{cat},3}^{\pm }\left(Z|l\right)$ coincide with the marginal conditional probability distributions shown in Table 2 for the case of two-qubit even and odd cat states; hence, they correspond to the mixed state in Equation (35).

Note that in the case of three qubits, it is possible to define marginal conditional probability distributions concerning any pairs of two qubits as

(45) $$\begin{array}{ccc} \tilde{w}\left(X,Y|j,k\right) & = & \sum_{Z}w\left(X,Y,Z|j,k,l\right), \end{array}$$

(46) $$\begin{array}{ccc} \tilde{w}\left(X,Z|j,l\right) & = & \sum_{Y}w\left(X,Y,Z|j,k,l\right), \end{array}$$

(47) $$\begin{array}{ccc} \tilde{w}\left(Y,Z|k,l\right) & = & \sum_{X}w\left(X,Y,Z|j,k,l\right). \end{array}$$

These expressions are valid for any values of *j*, *k*, and *l*. As an example, we show the components of the marginal conditional probability distribution ${\tilde{w}}_{\mathrm{cat},3}^{+}\left(X,Y|j,k\right)$ of three-qubit even cat states in Table A9. Note that the marginal conditional probability distributions ${\tilde{w}}_{\mathrm{cat},3}^{+}\left(X,Z|j,l\right)$ and ${\tilde{w}}_{\mathrm{cat},3}^{+}\left(Y,Z|k,l\right)$ coincide with the marginal conditional probability distribution ${\tilde{w}}_{\mathrm{cat},3}^{+}\left(X,Y|j,k\right)$. These probabilities correspond to the mixed state that can be obtained by taking the partial trace of the state (37) for any of the qubits, that is,

(48) $${\hat{\rho }}^{\pm }={\mathrm{Tr}}_{1}\left[{\hat{\rho }}_{\mathrm{cat},3}^{\pm }\right]={\mathrm{Tr}}_{2}\left[{\hat{\rho }}_{\mathrm{cat},3}^{\pm }\right]={\mathrm{Tr}}_{3}\left[{\hat{\rho }}_{\mathrm{cat},3}^{\pm }\right]=\frac{\left|00\rangle \langle 00|+|11\rangle \langle 11\right|}{2}.$$

One can easily check that the components in Table A9 satisfy the normalization condition in Equation (30). An unexpected property of this probability distribution is that, contrary to the case of the single-mode mixed state (35), not all the elements take the same value of 0.25 as the marginal probabilities for j=k=3 are 0.5 or 0. The zero values correspond to the impossible events that the spin measurements in the *z* direction for the two qubits lead to values with opposite signs; that is, obtaining +1/2 for the first qubit and −1/2 for the second qubit (and vice versa) simultaneously is impossible. This is due to the absence of the terms |01〉〈01| and |10〉〈10| in the mixed state of Equation (48).

Note that the two-qubit even cat state presented in Equation (27) is the superposition of the two pure states |00〉 and |11〉, while the mixed state in Equation (48) is the superposition of the two corresponding projectors |00〉〈00| and |11〉〈11|. By comparing their probability distributions presented in Table A1 and Table A9, one can deduce that the four zero-probabilities appearing for the measurements along the *x* and *y* directions in Table A1 but missing from Table A9 distinguish the entangled state (27) from the mixed state (48).

### 3.4. Cat States of an Oscillating Spin-1/2 Particle

Finally, we consider even and odd cat states of an oscillating spin-1/2 particle. These states are defined in the tensor product Hilbert space $H=H_{\mathrm{osc}}⊗H_{1/2}$ and they read as

(49) $$|\Psi_{\mathrm{cat},1/2}^{\pm }\rangle =\frac{1}{\sqrt{2}}(|\alpha \rangle ⊗|0\rangle \pm |-\alpha \rangle ⊗|1\rangle ).$$

Obviously, these are entangled states; hence, they cannot be written as the product of a spin-1/2 state and an oscillator state.

Using the dequantizer operator of continuous-variable quantum systems $\delta \left(X\hat{1}-\mu \hat{q}-\nu \hat{p}\right)$ and that of discrete-variable quantum systems $\hat{U}\left(Y|j\right)$, the density operator $\hat{\rho }$ of a state in the Hilbert space H can be mapped onto the function w(X,Y∣μ,ν,j) as

(50) $$w\left(X,Y|\mu ,\nu ,j\right)=\mathrm{Tr}\left[\hat{\rho }\;\delta \left(X\hat{1}-\mu \hat{q}-\nu \hat{p}\right)\hat{U}\left(Y|j\right)\right].$$

As w(X,Y∣μ,ν,j) is a nonnegative conditional probability distribution function, it satisfies the normalization condition:

(51) $$\int \sum_{Y}w\left(X,Y|\mu ,\nu ,j\right)\;dX=1.$$

To our knowledge, the probability representation of quantum states, which is a combination of continuous and discrete probability distributions, has not been discussed in the literature.

The marginal conditional probability distributions $\tilde{w}\left(Y|j\right)$ and $\tilde{w}\left(X|\mu ,\nu \right)$ determining the states of the spin and the oscillator, respectively, can be derived as

(52) $$\begin{array}{ccc} \tilde{w}\left(Y|j\right) & = & \int w(X,Y|\mu ,\nu ,j)\;dX, \end{array}$$

(53) $$\begin{array}{ccc} \tilde{w}\left(X|\mu ,\nu \right) & = & \sum_{Y}w\left(X,Y|\mu ,\nu ,j\right). \end{array}$$

The density operators of the even and odd cat states of an oscillating spin-1/2 particle reads

(54) $$\begin{array}{ccc} {\hat{\rho }}_{\mathrm{cat},1/2}^{\pm } & = & |\Psi_{\mathrm{cat},1/2}^{\pm }\rangle \langle \Psi_{\mathrm{cat},1/2}^{\pm }|\\ & = & \frac{1}{2}\left(|\alpha \rangle \langle \alpha |⊗|0\rangle \langle 0|\pm |\alpha \rangle \langle -\alpha |⊗|0\rangle \langle 1|\right.\\ & & \left.\pm |-\alpha \rangle \langle \alpha |⊗|1\rangle \langle 0|+|-\alpha \rangle \langle -\alpha |⊗|1\rangle \langle 1|\right). \end{array}$$

Using Equation (50), the conditional probability distribution $w_{\mathrm{cat},1/2}^{\pm }\left(X,Y|\mu ,\nu ,j\right)$ of even and odd cat states of an oscillating spin-1/2 particle can be written in the form

(55) $$\begin{array}{c} w_{\mathrm{cat},1/2}^{\pm }\left(X,Y|\mu ,\nu ,j\right)=\frac{1}{2}\left(w_{\left|\alpha \rangle \langle \alpha \right|}\left(X|\mu ,\nu \right)w_{\left|0\rangle \langle 0\right|}\left(Y|j\right)\pm w_{\left|\alpha \rangle \langle -\alpha \right|}\left(X|\mu ,\nu \right)w_{\left|0\rangle \langle 1\right|}\left(Y|j\right)\right.\\ \left.\pm w_{\left|-\alpha \rangle \langle \alpha \right|}\left(X|\mu ,\nu \right)w_{\left|1\rangle \langle 0\right|}\left(Y|j\right)+w_{\left|-\alpha \rangle \langle -\alpha \right|}\left(X|\mu ,\nu \right)w_{\left|1\rangle \langle 1\right|}\left(Y|j\right)\right). \end{array}$$

Note that the factors in the terms of this expression are not probability distributions in themselves. The factors $w_{\left|\pm \alpha \rangle \langle \pm \alpha \right|}\left(X|\mu ,\nu \right)$ can be derived in the following way:

(56) $$\begin{array}{c} w_{\left|\pm \alpha \rangle \langle \pm \alpha \right|}\left(X|\mu ,\nu \right)=\mathrm{Tr}\left[|\pm \alpha \rangle \langle \pm \alpha |\;\delta \left(X\hat{1}-\mu \hat{q}-\nu \hat{p}\right)\right]. \end{array}$$

Using the wavefunction of the coherent state,

(57) $$\phi_{\alpha }\left(y\right)=\frac{1}{\pi^{1/4}}\exp\left(-\frac{y^{2}}{2}+\sqrt{2}\alpha y-\frac{{\left|\alpha \right|}^{2}}{2}-\frac{\alpha^{2}}{2}\right)$$

and Equation (8), the factors $w_{\left|\pm \alpha \rangle \langle \pm \alpha \right|}\left(X|\mu ,\nu \right)$ can be calculated as

(58) w|±α〉〈±α|=12π|ν|12πexp(2Re(α)2))∫−∞∞exp2(±α)y−y22expiμy22ν−iXyνdy×∫−∞∞exp−2(±α*)y−y22exp−iμy22ν+iXyνdy.

Applying this formula and using the integral expression

(59) $$\int_{-\infty }^{\infty }\exp\left(\frac{1}{2}iax^{2}+iJx\right)dx={\left(\frac{2\pi i}{a}\right)}^{1/2}\exp\left(\frac{-iJ^{2}}{2a}\right),$$

we obtain

(60) w|α〉〈α|(X∣μ,ν)=Nexp[23/2X(μRe(α)+νIm(α))−X2μ2+ν2],

(61) w|α〉〈−α|(X∣μ,ν)=Nexp[i23/2X(μIm(α)−νRe(α))−X2μ2+ν2],

(62) w|−α〉〈α|(X∣μ,ν)=Nexp[−i23/2X(μIm(α)−νRe(α))−X2μ2+ν2],

(63) w|−α〉〈−α|(X∣μ,ν)=Nexp[−23/2X(μRe(α)+νIm(α))−X2μ2+ν2],

where

(64) N=1μ2+ν212πexp(2Re(α)2))exp−4μνIm(α)Re(α)+2ν2(Re(α)2−Im(α)2)μ2+ν2.

Next, we express the factors $w_{\left|0\rangle \langle 0\right|}\left(Y|j\right)$, $w_{\left|0\rangle \langle 1\right|}\left(Y|j\right)$, $w_{\left|1\rangle \langle 0\right|}\left(Y|j\right)$, $w_{\left|1\rangle \langle 1\right|}\left(Y|j\right)$ in Equation (55). These factors can be written as

(65) $$\begin{array}{c} w_{\left|0\rangle \langle 0\right|}\left(Y|j\right)=\mathrm{Tr}(|0\rangle \langle 0|\hat{U}\left(Y|j\right)), \end{array}$$

(66) $$\begin{array}{c} w_{\left|0\rangle \langle 1\right|}\left(Y|j\right)=\mathrm{Tr}(|0\rangle \langle 1|\hat{U}\left(Y|j\right)), \end{array}$$

(67) $$\begin{array}{c} w_{\left|1\rangle \langle 0\right|}\left(Y|j\right)=\mathrm{Tr}(|1\rangle \langle 0|\hat{U}\left(Y|j\right)), \end{array}$$

(68) $$\begin{array}{c} w_{\left|1\rangle \langle 1\right|}\left(Y|j\right)=\mathrm{Tr}(|1\rangle \langle 1|\hat{U}\left(Y|j\right)), \end{array}$$

where the dequantizer operators $\hat{U}\left(Y|j\right)$ are given in Equation (21). The results are given in Table 3.

Recall that the factors presented in Table 3 are not probability distributions in themselves. As a consequence, negative or complex numbers occur in the table in the case of the cross-factors $w_{\left|0\rangle \langle 1\right|}\left(Y|j\right)$ and $w_{\left|1\rangle \langle 0\right|}\left(Y|j\right)$. The components of the conditional probability distribution $w_{\mathrm{cat},1/2}^{\pm }\left(X,Y|\mu ,\nu ,j\right)$ can be calculated using the factors in Table 3 and in Equations (60)–(64).

Next, we determine the marginal conditional probability distributions ${\tilde{w}}_{\mathrm{cat},1/2}^{\pm }\left(Y|j\right)$ defined in Equation (52). First, we calculate the integrals

(69) $$\int w_{\left|\pm \alpha \rangle \langle \pm \alpha \right|}\left(X|\mu ,\nu \right)\;dX$$

by using Equations (60)–(63) and the integral expression (59). Next, by applying the factors presented in Table 3, we obtain the components of the marginal conditional probability distribution presented in Table 4.

Note that some components of the marginal conditional probability distribution ${\tilde{w}}_{\mathrm{cat},1/2}^{\pm }\left(Y|j\right)$ still contain the parameter α, but this dependence tends to zero as α→0. In this limit, the coherent states |α〉 and |−α〉 become orthogonal to each other. Then, the marginal conditional probability distribution ${\tilde{w}}_{\mathrm{cat},1/2}^{\pm }\left(Y|j\right)$ corresponds to the mixed state in Equation (35).

Finally, we derive the marginal conditional probability distribution ${\tilde{w}}_{\mathrm{cat},1/2}^{\pm }\left(X|\mu ,\nu \right)$ using Equation (53). Using the factors presented in Equations (60)–(64) and in Table 3, we obtain

(70) w˜cat,1/2±(X∣μ,ν)=12w|α〉〈α|(X∣μ,ν)+w|−α〉〈−α|(X∣μ,ν)=121μ2+ν212πexp(2Re(α)2))exp−4μνIm(α)Re(α)+2ν2(Re(α)2−Im(α)2)μ2+ν2exp[23/2X(μRe(α)+νIm(α))−X2μ2+ν2]+exp[−23/2X(μRe(α)+νIm(α))−X2μ2+ν2].

Note that these probability distributions are the same for even and odd cat states. These probabilities correspond to the mixed state that can be obtained by taking the partial trace of the state (49) for the state of the 1/2 spin; that is,

(71) $$\begin{array}{ccc} {\hat{\rho }}^{\pm } & = & {\mathrm{Tr}}_{1/2}\left[|\Psi_{\mathrm{cat},1/2}^{\pm }\rangle \langle \Psi_{\mathrm{cat},1/2}^{\pm }|\right]=\frac{1}{2}\left(|\alpha \rangle \langle \alpha |+|-\alpha \rangle \langle -\alpha |\right). \end{array}$$

These mixed states are composed of two projectors on coherent states with the same amplitudes but opposite signs with equal weights.

## 4. Conclusions

We have considered the probability representations of even and odd cat states of two and three qubits. These states are even and odd superpositions of spin-1/2 eigenstates corresponding to opposite directions along the *z*-axis for the particular qubits. They are entangled states, and, in the case of two qubits, they correspond to two of the Bell states, while for three qubits the even cat state is equal to the GHZ state. The components of the derived probability distributions correspond to the probabilities of various spin projections onto the opposite directions of the perpendicular *x*, *y*, and *z* axes for the particular qubit. Accordingly, these components can be measured in experiments by repeated spin projection measurements using a sufficiently large set of identically prepared states.

We have determined the values of the components of the entangled conditional probability distributions of two-qubit even and odd cat states and those of three-qubit even cat states. We have also derived the components of the marginal conditional probability distributions concerning the states of the particular qubits for all these states. These marginal probability distributions are the same for even and odd cat states, and they correspond to the mixed state of a single qubit, which is composed as a sum of projectors on orthogonal spin states with equal weights. We have also derived the components of the marginal conditional probability distributions concerning any pairs of two qubits for the three-qubit even cat state. These marginal probability distributions are the same for even and odd cat states, and they correspond to the mixed state of two qubits, which is composed as a sum of projectors on orthogonal spin states with equal weights for both qubits. We have analyzed the properties of the entangled probability distributions of the considered cat states, and we have shown how the entanglement appears in the components of the distributions. We have also discussed the difference between the probability representation of two-qubit even cat state and that of the two-qubit mixed states that can be obtained by taking the partial trace for any of the qubits of the three-qubit even cat state. We have found that certain zero-valued components of the distribution corresponding to the impossible spin-measurement outputs that appear in the probability distribution of the entangled pure state but are not present in the distribution of the mixed state can identify the entangled cat state.

Finally, we have considered the probability representation of even and odd cat states of an oscillating spin-1/2 particle. We have introduced an entangled conditional probability distribution with both a continuous and a discrete variable concerning the oscillator and the spin-1/2 states, respectively. We have derived all the factors required to determine the entangled conditional probability distributions describing the density matrices of even and odd cat states of an oscillating spin-1/2 particle. We have also defined the marginal conditional probability distributions characterizing the state of either the spin or the oscillator, and we have determined these distributions. The developed approach to derive the probability representations of cat states of qubit systems can be generalized for the case of qudit cat states (qutrits, ququarts); this provides the possibility to extend the concept of entangled probability distributions that are not available in contemporary probability theory.

## Figures and Tables

**Table 1:** The conditional probabilities w(X∣j) obtained for qubit even and odd cat states.

X∣j	$w_{\mathrm{cat},1}^{+}\left(X|j\right)$	$w_{\mathrm{cat},1}^{-}\left(X|j\right)$
+1/2|1	1	0
+1/2|2	0.5	0.5
+1/2|3	0.5	0.5
−1/2|1	0	1
−1/2|2	0.5	0.5
−1/2|3	0.5	0.5

**Table 2:** The components of the marginal conditional probability distributions ${\tilde{w}}_{\mathrm{cat},2}^{\pm }\left(X|j\right)$ and ${\tilde{w}}_{\mathrm{cat},2}^{\pm }\left(Y|k\right)$ of two-qubit even and odd cat states shown in Equation (28).

X∣j or Y∣k	${\tilde{w}}_{\mathrm{cat},2}^{\pm }\left(X|j\right)$	${\tilde{w}}_{\mathrm{cat},2}^{\pm }\left(Y|k\right)$
+1/2|1	0.5	0.5
+1/2|2	0.5	0.5
+1/2|3	0.5	0.5
−1/2|1	0.5	0.5
−1/2|2	0.5	0.5
−1/2|3	0.5	0.5

**Table 3:** The factors $w_{\left|0\rangle \langle 0\right|}\left(Y|j\right)$, $w_{\left|0\rangle \langle 1\right|}\left(Y|j\right)$, $w_{\left|1\rangle \langle 0\right|}\left(Y|j\right)$, $w_{\left|1\rangle \langle 1\right|}\left(Y|j\right)$ appearing in the conditional probability distribution w(X,Y∣μ,ν,j) of even and odd cat states of an oscillating spin-1/2 particle displayed in Equation (55). The rows of the table correspond to various pairs of the parameters *Y* and *j*, while the values of the factors are presented in the columns.

Y∣j	$w_{\left|0\rangle \langle 0\right|}\left(Y|j\right)$	$w_{\left|0\rangle \langle 1\right|}\left(Y|j\right)$	$w_{\left|1\rangle \langle 0\right|}\left(Y|j\right)$	$w_{\left|1\rangle \langle 1\right|}\left(Y|j\right)$
+1/2∣ 1	0.5	0.5	0.5	0.5
+1/2∣ 2	0.5	0.5i	−0.5i	0.5
+1/2∣ 3	1	0	0	0
−1/2∣ 1	0.5	−0.5	−0.5	0.5
−1/2∣ 2	0.5	−0.5i	0.5i	0.5
−1/2∣ 3	0	0	0	1

**Table 4:** The components of the marginal conditional probability distribution ${\tilde{w}}_{\mathrm{cat},1/2}^{\pm }\left(Y|j\right)$ of even and odd cat states of an oscillating spin-1/2 particle are shown in Equation (55).

Y∣j	$\tilde{w}\left(Y|j\right)$
+1/2∣ 1	0.5$\left[1\pm \exp(-2|\alpha {|}^{2})\right]$
+1/2∣ 2	0.5
+1/2∣ 3	0.5
−1/2∣ 1	0.5$\left[1\mp \exp(-2|\alpha {|}^{2})\right]$
−1/2∣ 2	0.5
−1/2∣ 3	0.5

## Data Availability

Data underlying the results presented in this paper are not publicly available at this time but may be obtained from the authors upon reasonable request.

## Appendix A

In the Appendix, we present the conditional probability distributions determined for even and odd cat states for two qubits and for even cat states for three qubits, and the marginal conditional probability distribution is determined for the three-qubit even cat state.

First, we present the components of the conditional probability distributions $w_{\mathrm{cat},2}^{\pm }\left(X,Y|j,k\right)$ of the even and odd cat states for two qubits $|\Phi_{\mathrm{cat},2}^{\pm }\rangle$.

**Table A1:** Components of the conditional probability distribution $w_{\mathrm{cat},2}^{+}\left(X,Y|j,k\right)$ of the even cat state for two qubits $|\Phi_{\mathrm{cat},2}^{+}\rangle$. The rows of the table correspond to various pairs of the parameters *X* and *j*, while the columns of the table correspond to various pairs of the parameters *Y* and *k*.

	Y∣k	+1/2∣1	+1/2∣2	+1/2∣3	−1/2∣1	−1/2∣2	−1/2∣3
X∣j							
+1/2∣1		0.5	0.25	0.25	0	0.25	0.25
+1/2∣2		0.25	0	0.25	0.25	0.5	0.25
+1/2∣3		0.25	0.25	0.5	0.25	0.25	0
−1/2∣1		0	0.25	0.25	0.5	0.25	0.25
−1/2∣2		0.25	0.5	0.25	0.25	0	0.25
−1/2∣3		0.25	0.25	0	0.25	0.25	0.5

**Table A2:** Components of the conditional probability distribution $w_{\mathrm{cat},2}^{-}\left(X,Y|j,k\right)$ of the odd cat state for two qubits $|\Phi_{\mathrm{cat},2}^{-}\rangle$. The rows of the table correspond to various pairs of the parameters *X* and *j*, while the columns of the table correspond to various pairs of the parameters *Y* and *k*.

	Y∣k	+1/2∣1	+1/2∣2	+1/2∣3	−1/2∣1	−1/2∣2	−1/2∣3
X∣j							
+1/2|1		0	0.25	0.25	0.5	0.25	0.25
+1/2|2		0.25	0.5	0.25	0.25	0	0.25
+1/2|3		0.25	0.25	0.5	0.25	0.25	0
−1/2|1		0.5	0.25	0.25	0	0.25	0.25
−1/2|2		0.25	0	0.25	0.25	0.5	0.25
−1/2|3		0.25	0.25	0	0.25	0.25	0.5

In Table A3, Table A4, Table A5, Table A6, Table A7 and Table A8, we show the components of the conditional probability distribution $w_{\mathrm{cat},3}^{+}\left(X,Y,Z|j,k,l\right)$ of the even cat states for three qubits $|\Psi_{\mathrm{cat},3}^{+}\rangle$.

**Table A3:** Components of the conditional probability distribution $w_{\mathrm{cat},3}^{+}\left(X,Y,Z|j,k,l\right)$ of the even cat state for three qubits $|\Psi_{\mathrm{cat},3}^{+}\rangle$ for X=+1/2 and j=1. The rows of the table correspond to various pairs of the parameters *Y* and *k*, while the columns of the table correspond to various pairs of the parameters *Z* and *l*.

	Z∣l	+1/2∣1	+1/2∣2	+1/2∣3	−1/2∣1	−1/2∣2	−1/2∣3
Y∣k							
+1/2|1		0.25	0.125	0.125	0	0.125	0.125
+1/2|2		0.125	0	0.125	0.125	0.25	0.125
+1/2|3		0.125	0.125	0.25	0.125	0.125	0
−1/2|1		0	0.125	0.125	0.25	0.125	0.125
−1/2|2		0.125	0.25	0.125	0.125	0	0.125
−1/2|3		0.125	0.125	0	0.125	0.125	0.25

**Table A4:** Components of the conditional probability distribution $w_{\mathrm{cat},3}^{+}\left(X,Y,Z|j,k,l\right)$ of the even cat state for three qubits $|\Psi_{\mathrm{cat},3}^{+}\rangle$ for X=+1/2 and j=2. The rows of the table correspond to various pairs of the parameters *Y* and *k*, while the columns of the table correspond to various pairs of the parameters *Z* and *l*.

	Z∣l	+1/2∣1	+1/2∣2	+1/2∣3	−1/2∣1	−1/2∣2	−1/2∣3
Y∣k							
+1/2|1		0.125	0	0.125	0.125	0.25	0.125
+1/2|2		0	0.125	0.125	0.25	0.125	0.125
+1/2|3		0.125	0.125	0.25	0.125	0.125	0
−1/2|1		0.125	0.25	0.125	0.125	0	0.125
−1/2|2		0.25	0.125	0.125	0	0.125	0.125
−1/2|3		0.125	0.125	0	0.125	0.125	0.25

**Table A5:** Components of the conditional probability distribution $w_{\mathrm{cat},3}^{+}\left(X,Y,Z|j,k,l\right)$ of the even cat state for three qubits $|\Psi_{\mathrm{cat}.3}^{+}\rangle$ for X=+1/2 and j=3. The rows of the table correspond to various pairs of the parameters *Y* and *k*, while the columns of the table correspond to various pairs of the parameters *Z* and *l*.

	Z∣l	+1/2∣1	+1/2∣2	+1/2∣3	−1/2∣1	−1/2∣2	−1/2∣3
Y∣k							
+1/2|1		0.125	0.125	0.25	0.125	0.125	0
+1/2|2		0.125	0.125	0.25	0.125	0.125	0
+1/2|3		0.25	0.25	0.5	0.25	0.25	0
−1/2|1		0.125	0.125	0.25	0.125	0.125	0
−1/2|2		0.125	0.125	0.25	0.125	0.125	0
−1/2|3		0	0	0	0	0	0

**Table A6:** Components of the conditional probability distribution $w_{\mathrm{cat},3}^{+}\left(X,Y,Z|j,k,l\right)$ of the even cat state for three qubits $|\Psi_{\mathrm{cat},3}^{+}\rangle$ for X=−1/2 and j=1. The rows of the table correspond to various pairs of the parameters *Y* and *k*, while the columns of the table correspond to various pairs of the parameters *Z* and *l*.

	Z∣l	+1/2∣1	+1/2∣2	+1/2∣3	−1/2∣1	−1/2∣2	−1/2∣3
Y∣k							
+1/2|1		0	0.125	0.125	0.25	0.125	0.125
+1/2|2		0.125	0.25	0.125	0.125	0	0.125
+1/2|3		0.125	0.125	0.25	0.125	0.125	0
−1/2|1		0.25	0.125	0.125	0	0.125	0.125
−1/2|2		0.125	0	0.125	0.125	0.25	0.125
−1/2|3		0.125	0.125	0	0.125	0.125	0.25

**Table A7:** Components of the conditional probability distribution $w_{\mathrm{cat},3}^{+}\left(X,Y,Z|j,k,l\right)$ of the even cat state for three qubits $|\Psi_{\mathrm{cat},3}^{+}\rangle$ for X=−1/2 and j=2. The rows of the table correspond to various pairs of the parameters *Y* and *k*, while the columns of the table correspond to various pairs of the parameters *Z* and *l*.

	Z∣l	+1/2∣1	+1/2∣2	+1/2∣3	−1/2∣1	−1/2∣2	−1/2∣3
Y∣k							
+1/2|1		0.125	0.25	0.125	0.125	0	0.125
+1/2|2		0.25	0.125	0.125	0	0.125	0.125
+1/2|3		0.125	0.125	0.25	0.125	0.125	0
−1/2|1		0.125	0	0.125	0.125	0.25	0.125
−1/2|2		0	0.125	0.125	0.25	0.125	0.125
−1/2|3		0.125	0.125	0	0.125	0.125	0.25

**Table A8:** Components of the conditional probability distribution $w_{\mathrm{cat},3}^{+}\left(X,Y,Z|j,k,l\right)$ of the even cat state for three qubits $|\Psi_{\mathrm{cat},3}^{+}\rangle$ for X=−1/2 and j=3. The rows of the table correspond to various pairs of the parameters *Y* and *k*, while the columns of the table correspond to various pairs of the parameters *Z* and *l*.

	Z∣l	+1/2∣1	+1/2∣2	+1/2∣3	−1/2∣1	−1/2∣2	−1/2∣3
Y∣k							
+1/2|1		0.125	0.125	0	0.125	0.125	0.25
+1/2|2		0.125	0.125	0	0.125	0.125	0.25
+1/2|3		0	0	0	0	0	0
−1/2|1		0.125	0.125	0	0.125	0.125	0.25
−1/2|2		0.125	0.125	0	0.125	0.125	0.25
−1/2|3		0.25	0.25	0	0.25	0.25	0.5

Finally, we present the components of the marginal conditional probability distribution ${\tilde{w}}_{\mathrm{cat},3}^{+}\left(X,Y|j,k\right)$ of the three-qubit even cat state $|\Psi_{\mathrm{cat},3}^{+}\rangle$.

**Table A9:** Components of the marginal conditional probability distribution ${\tilde{w}}_{\mathrm{cat},3}^{+}\left(X,Y|j,k\right)$ of the three-qubit even cat state $|\Psi_{\mathrm{cat},3}^{+}\rangle$ shown in Equation (36).

	Y∣k	+1/2∣1	+1/2∣2	+1/2∣3	−1/2∣1	−1/2∣2	−1/2∣3
X∣j							
+1/2|1		0.25	0.25	0.25	0.25	0.25	0.25
+1/2|2		0.25	0.25	0.25	0.25	0.25	0.25
+1/2|3		0.25	0.25	0.5	0.25	0.25	0
−1/2|1		0.25	0.25	0.25	0.25	0.25	0.25
−1/2|2		0.25	0.25	0.25	0.25	0.25	0.25
−1/2|3		0.25	0.25	0	0.25	0.25	0.5

## References

[1] Mancini S., Man’ko V.I., Tombesi P. (1996). Symplectic tomography as classical approach to quantum systems. Phys. Lett. A.

[2] Dodonov V., Man’ko V. (1997). Positive distribution description for spin states. Phys. Lett. A.

[3] Man’ko V.I., Man’ko O.V. (1997). Spin state tomography. J. Exp. Theor. Phys..

[4] Man’ko O. (1998). Tomography of spin states and classical formulation of quantum mechanics. Symmetries in Science X.

[5] Ibort A., Man’ko V.I., Marmo G., Simoni A., Ventriglia F. (2009). An introduction to the tomographic picture of quantum mechanics. Phys. Scr..

[6] Amosov G.G., Korennoy Y.A., Man’ko V.I. (2012). Description and measurement of observables in the optical tomographic probability representation of quantum mechanics. Phys. Rev. A.

[7] Asorey M., Ibort A., Marmo G., Ventriglia F. (2015). Quantum Tomography twenty years later. Phys. Scr..

[8] Man’ko O.V., Man’ko V.I. (2021). Probability Representation of Quantum States. Entropy.

[9] Glauber R.J. (1963). Photon Correlations. Phys. Rev. Lett..

[10] Sudarshan E.C.G. (1963). Equationivalence of Semiclassical and Quantum Mechanical Descriptions of Statistical Light Beams. Phys. Rev. Lett..

[11] Husimi K. (1940). Some Formal Properties of the Density Matrix. Proc. Phys.-Math. Soc. Jpn..

[12] Kano Y. (1965). A New Phase-Space Distribution Function in the Statistical Theory of the Electromagnetic Field. J. Math. Phys..

[13] Wigner E. (1932). On the Quantum Correction For Thermodynamic Equationilibrium. Phys. Rev..

[14] Man’ko V.I., Markovich L.A. (2020). Integral transforms between tomogram and quasi-probability functions based on quantizer-dequantizer operators formalism. J. Math. Phys..

[15] Man’ko O.V., Man’ko V.I., Marmo G. (2002). Alternative commutation relations, star products and tomography. J. Phys. A Math. Gen..

[16] Smithey D.T., Beck M., Raymer M.G., Faridani A. (1993). Measurement of the Wigner distribution and the density matrix of a light mode using optical homodyne tomography: Application to squeezed states and the vacuum. Phys. Rev. Lett..

[17] Bertrand J., Bertrand P. (1987). A tomographic approach to Wigner’s function. Found. Phys..

[18] Vogel K., Risken H. (1989). Determination of quasiprobability distributions in terms of probability distributions for the rotated quadrature phase. Phys. Rev. A.

[19] D’Ariano G.M., Leonhardt U., Paul H. (1995). Homodyne detection of the density matrix of the radiation field. Phys. Rev. A.

[20] Mauro D’Ariano G., Paris M.G., Sacchi M.F. (2003). Quantum Tomography. Adv. Imag. Elect. Phys..

[21] Lvovsky A.I., Raymer M.G. (2009). Continuous-variable optical quantum-state tomography. Rev. Mod. Phys..

[22] Man’ko M.A., Man’ko V.I. (2023). Quantum Oscillator at Temperature T and the Evolution of a Charged-Particle State in the Electric Field in the Probability Representation of Quantum Mechanics. Entropy.

[23] Ádám P., Man’ko M.A., Man’ko V.I. (2022). Even and Odd Schrödinger Cat States in the Probability Representation of Quantum Mechanics. J. Russ. Laser Res..

[24] Man’ko O.V., Man’ko V.I. (2023). Inverted Oscillator Quantum States in the Probability Representation. Entropy.

[25] Mechler M., Man’ko M.A., Man’ko V.I., Adam P. (2024). Probability Representation of Nonclassical States of the Inverted Oscillator. J. Russ. Laser Res..

[26] Chernega V.N., Man’ko O.V. (2023). Dynamics of System States in the Probability Representation of Quantum Mechanics. Entropy.

[27] Cunha M.O.T., Man’ko V.I., Scully M.O. (2001). Quasiprobability and Probability Distributions for Spin-1/2 States. Found. Phys. Lett..

[28] D’Ariano G.M., Maccone L., Paini M. (2003). Spin tomography. J. Opt. B Quantum Semiclass. Opt..

[29] Adam P., Andreev V.A., Man’ko M.A., Man’ko V.I., Mechler M. (2020). Star-Product Formalism for the Probability and Mean-Value Representations of Qudits. J. Russ. Laser Res..

[30] Adam P., Andreev V.A., Man’ko M.A., Man’ko V.I., Mechler M. (2021). Properties of Quantizer and Dequantizer Operators for Qudit States and Parametric Down-Conversion. Symmetry.

[31] Chernega V.N., Man’ko O.V., Man’ko V.I. (2022). Entangled Qubit States and Linear Entropy in the Probability Representation of Quantum Mechanics. Entropy.

[32] Man’ko M.A., Man’ko V.I. (2023). Probability Distributions Describing Qubit-State Superpositions. Entropy.

[33] Bell J.S. (1964). On the Einstein Podolsky Rosen paradox. Phys. Phys. Fiz..

[34] Bell J.S., Aspect A. (2004). Speakable and Unspeakable in Quantum Mechanics: Collected Papers on Quantum Philosophy.

[35] Kálmán O., Gábris A., Jex I., Kiss T. (2024). Unambiguous preparation of Bell pairs. arXiv.

[36] Greenberger D.M., Horne M.A., Zeilinger A. (1993). Multiparticle Interferometry and the Superposition Principle. Phys. Today.

[37] Cao H., Hansen L.M., Giorgino F., Carosini L., Zahálka P., Zilk F., Loredo J.C., Walther P. (2024). Photonic Source of Heralded Greenberger-Horne-Zeilinger States. Phys. Rev. Lett..

[38] Ekert A.K. (1991). Quantum cryptography based on Bell’s theorem. Phys. Rev. Lett..

[39] Bennett C.H., Brassard G., Crépeau C., Jozsa R., Peres A., Wootters W.K. (1993). Teleporting an unknown quantum state via dual classical and Einstein-Podolsky-Rosen channels. Phys. Rev. Lett..

[40] Nickerson N.H., Li Y., Benjamin S.C. (2013). Topological quantum computing with a very noisy network and local error rates approaching one percent. Nat. Commun..

[41] Monroe C., Raussendorf R., Ruthven A., Brown K.R., Maunz P., Duan L.M., Kim J. (2014). Large-scale modular quantum-computer architecture with atomic memory and photonic interconnects. Phys. Rev. A.

[42] Li Z., Jiang X., Liu L. (2022). Multi-Party Quantum Secret Sharing Based on GHZ State. Entropy.

[43] Bužek V., Vidiella-Barranco A., Knight P.L. (1992). Superpositions of coherent states: Squeezing and dissipation. Phys. Rev. A.

[44] Dodonov V.V., Man’ko V.I., Nikonov D.E. (1995). Even and odd coherent states for multimode parametric systems. Phys. Rev. A.

[45] Agarwal G., Puri R., Singh R. (1997). Atomic Schrödinger cat states. Phys. Rev. A.

[46] Brune M., Hagley E., Dreyer J., Maître X., Maali A., Wunderlich C., Raimond J.M., Haroche S. (1996). Observing the Progressive Decoherence of the “Meter” in a Quantum Measurement. Phys. Rev. Lett..

[47] Auffeves A., Maioli P., Meunier T., Gleyzes S., Nogues G., Brune M., Raimond J.M., Haroche S. (2003). Entanglement of a Mesoscopic Field with an Atom Induced by Photon Graininess in a Cavity. Phys. Rev. Lett..

[48] Neergaard-Nielsen J.S., Nielsen B.M., Hettich C., Mølmer K., Polzik E.S. (2006). Generation of a Superposition of Odd Photon Number States for Quantum Information Networks. Phys. Rev. Lett..

[49] Ourjoumtsev A., Jeong H., Tualle-Brouri R., Grangier P. (2007). Generation of optical ‘Schrödinger cats’ from photon number states. Nature.

[50] Takahashi H., Wakui K., Suzuki S., Takeoka M., Hayasaka K., Furusawa A., Sasaki M. (2008). Generation of Large-Amplitude Coherent-State Superposition via Ancilla-Assisted Photon Subtraction. Phys. Rev. Lett..

[51] He B., Nadeem M., Bergou J.A. (2009). Scheme for generating coherent-state superpositions with realistic cross-Kerr nonlinearity. Phys. Rev. A.

[52] Adam P., Darázs Z., Kiss T., Mechler M. (2011). Double self-Kerr scheme for optical Schrödinger-cat state preparation. Phys. Scr..

[53] Lee C.W., Lee J., Nha H., Jeong H. (2012). Generating a Schrödinger-cat-like state via a coherent superposition of photonic operations. Phys. Rev. A.

[54] Bild M., Fadel M., Yang Y., von Lüpke U., Martin P., Bruno A., Chu Y. (2023). Schrödinger cat states of a 16-microgram mechanical oscillator. Science.

[55] Cochrane P.T., Milburn G.J., Munro W.J. (1999). Macroscopically distinct quantum-superposition states as a bosonic code for amplitude damping. Phys. Rev. A.

[56] Mirrahimi M. (2016). Cat-qubits for quantum computation. Comptes Rendus Phys..

[57] Grimm A., Frattini N.E., Puri S., Mundhada S.O., Touzard S., Mirrahimi M., Girvin S.M., Shankar S., Devoret M.H. (2020). Stabilization and operation of a Kerr-cat qubit. Nature.

[58] Su Q.P., Zhang Y., Yang C.P. (2022). Single-step implementation of a hybrid controlled-not gate with one superconducting qubit simultaneously controlling multiple target cat-state qubits. Phys. Rev. A.

[59] van Enk S., Hirota O. (2001). Entangled coherent states: Teleportation and decoherence. Phys. Rev. A.

[60] Jeong H., Kim M.S., Lee J. (2001). Quantum-information processing for a coherent superposition state via a mixedentangled coherent channel. Phys. Rev. A.

